# An Adaptive Task-Related Component Analysis Method for SSVEP Recognition

**DOI:** 10.3390/s22207715

**Published:** 2022-10-11

**Authors:** Vangelis P. Oikonomou

**Affiliations:** Information Technologies Institute, Centre for Research and Technology Hellas, Thermi-Thessaloniki, 57001 Thessaloniki, Greece; viknmu@iti.gr

**Keywords:** steady-state visual evoked potentials, EEG, task-related component analysis, multitask learning, spatial filtering, brain–computer interfaces

## Abstract

Steady-State Visual Evoked Potential (SSVEP) recognition methods use a subject’s calibration data to differentiate between brain responses, hence, providing the SSVEP-based brain–computer interfaces (BCIs) with high performance. However, they require sufficient calibration EEG trials to achieve that. This study develops a new method to learn from limited calibration EEG trials, and it proposes and evaluates a novel adaptive data-driven spatial filtering approach for enhancing SSVEP detection. The spatial filter learned from each stimulus utilizes temporal information from the corresponding EEG trials. To introduce the temporal information into the overall procedure, a multitask learning approach, based on the Bayesian framework, is adopted. The performance of the proposed method was evaluated into two publicly available benchmark datasets, and the results demonstrated that our method outperformed competing methods by a significant margin.

## 1. Introduction

A Brain–Computer Interface (BCI) is a device able to translate human brain activity into control signals, giving us an additional communication medium, other than physical. This device can be used to help people with motor disabilities [1], to augment communication abilities of healthy individuals [2], for entertainment [2], and, for neuromarketing purposes [3]. Brain activity can be measured with various specialized devices such as MRI scanners and electroencephalograms (EEG)-based devices. EEG devices are widely used since the required equipment is simple and inexpensive. EEG-based BCI systems utilize various brain responses such as motor imagery and visual responses from which Steady-State Visual Evoked Potentials (SSVEPs) are the more interesting of the brain’s responses since their usage results in minimal training requirements for the end-user and a higher Information Transfer Rate (ITR) than other similar systems [4,5]. When an individual is looking into a visual stimulus, which is flashing at fixed frequency, then a brain response is revealed in the occipital and occipital–parietal areas of the individual’s brain, which is called the SSVEP response [6]. An SSVEP response contains sinusoidal components which are related to the fundamental frequency of the visual stimulus as well as its harmonics. The overarching goal of a SSVEP BCI system is to detect the different frequency components corresponding to the visual stimuli and translate them into commands by using an EEG-based pattern recognition algorithms. SSVEP BCI systems have been used to develop assistive technologies, such as robotic wheelchairs [7] and robotic exoskeletons [8], as well as for communication and computer interaction [9,10], biometrics [11], emotion recognition [12], and entertainment [13]. Hence, the recognition of SSVEP signals in natural and noisy environments represents a significant problem.

The recognition of SSVEP responses involves the use of Machine Learning (ML) algorithms. Linear classifiers such as Support Vector Machines (SVMs) and Linear Discriminant Analysis (LDA) have been used to detect SSVEPs [9,14]. In addition, in [15], the use of Multivariate Linear Regression (MLR) was proposed to learn discriminative features for improving SSVEP classification, while in [16], kernel–based extensions of MLR were proposed using SSVEP-related kernels as an integral part of the Sparse Bayesian Learning (SBL) framework. Furthermore, Deep Learning (DL) approaches using Convolutional Neural Networks (CNN), based on time frequency analysis, are used to discriminate SSVEP responses [17,18,19]. However, DL approaches need SSVEP trials with large time windows to train the overall model, resulting in poor ITR performance.

SSVEP responses present specific frequency and spatial characteristics; hence, methods possessing these characteristics have been proposed. Among the first methods utilizing the above SSVEP characteristics is the Canonical Correlation Analysis (CCA) method [20]. The CCA uses sinusoids waves as reference templates, and it solves an optimization problem, based on multi-channel SSVEP data, in order to obtain optimal spatial filters. Extensions of CCA have been proposed in [21,22,23,24,25,26], extracting the subject-specific and task-related information from the individual calibration data and reducing the effect of spontaneous background EEG activities. From spatial filtering methods, the task-related component analysis (TRCA)-based method [24] shows great potential since it has achieved superior performance among various spatial filtering methods. The core idea of TRCA is to acquire the spatial filters by strengthening the task-related SSVEP components and suppressing the noise. TRCA-based methods are followed by the target detection step, where the similarity between the filtered test signal and the filtered template is calculated via the correlation coefficient. All spatial-filtering-based methods are based on the basic (generalized) eigenvalue problem [27,28]. However, the difference between various approaches can be observed, and these differences are reflected in the way that the matrices, involved in the eigenvalue problem, are constructed [28]. In [29], Correlated Component Analysis (CORCA) assumes that the task-related component is shared among the subjects by adopting a transfer learning procedure in the construction of covariance matrices. Meanwhile, in [30], a task-discriminant component analysis was applied, which involves the construction of within and between SSVEP target covariance matrices.

Recently, SSVEP BCI systems using ear-EEG recordings have been proposed [31,32]; however, the accuracy of such systems is low compare to classical approaches because EEG sensors are located far from the occipital cortex, resulting in noisy SSVEP signals. Furthermore, attempts to acquire trustworthy SSVEP signals using consumer-grade EEG devices have been increased [9,13,31,33,34]. Consumer-grade EEG devices provide us with EEG devices that more comfortable and have a better user experiences and applications in the real world. However, the quality of acquired EEG data is questionable, particularly due to the very complex and noisy environment in which they are acquired. Furthermore, fatigue caused by continuous visual stimulation can lead to user’s discomfort and degrade the system’s performance. A trade-off between signal quality, convenience, and user comfort has to be made in real practice. All the above indicate that the transition from laboratory experiments to real-world applications considerably affects the system performance. Hence, there is a need for new SSVEP recognition algorithms working in noisy environments.

In this article, we propose a new spatial-filtering-based method to extend basic ideas provided by the TRCA method. The TRCA only deals with limited noise components [35,36]. Additionally, its performance deteriorates drastically if the number of calibration trials are insufficient. To deal with more general noises and smaller numbers of trials, we introduce a novel adaptive time-domain filter resulting in more reliable similarity measurement. By introducing the temporally based filter into the objective function of the TRCA-based method, we construct a time filter that acts together with the spatial filter to suppress more general noises. Furthermore, the filter adapts to the statistical properties of SSVEP trials. Additionally, we test and compare our method with state-of-the-art (SoTA) approaches using two well-known, publicly available SSVEP datasets. The usage of these particular datasets give us the ability to check the performance of methods in different scenarios and conditions, approaching real-life experimental conditions. EEG recording in a mobile environment can cause artifacts and signal distortion, resulting in loss of accuracy and signal quality. Hence, one of the used SSVEP datasets has been recorded using an EPOC EEG mobile device. Furthermore, a short calibration of a BCI system, described as few-shot EEG learning [37], which uses minimal training data, is a major challenge in the BCI community. Hence, in our study, we provide experiments with respect to this issue. More specifically, we conducted experiments using a small number of EEG trials and EEG channels. Finally, we provide experiments using only channels that are placed in the prefrontal and temporal (i.e., hairless) brain regions.

The rest of this paper is organized as follows. In Section 2, we first provide a short description of CCA and TRCA concentrating on the their mathematical formulations, and then we describe our approach for SSVEP recognition. Section 3, we describe the SSVEP datasets that are used in our study, and then we provide details about our experiments and the performance of our method. In addition, a comparison with competing methods is provided. Finally, a short discussion and some concluding remarks are provided in Section 4.

## 2. Materials and Methods

### 2.1. Problem Description

When an SSVEP experiment is taking place, the subject is seated in front of a screen where visual stimuli are flashing in different frequencies. During the experiment, raw EEG data are collected in order to calibrate the overall system. The segmentation of raw EEG data (using event triggers) results in a set of trials for each visual stimulus (or class). Using these EEG trials, the experimenter can calibrate the BCI system (for example, by training the classifier). Let us assume that the SSVEP dataset is a collection of multi-channel EEG trials ${\left\{X_{1}^{\left(s\right)},X_{2}^{\left(s\right)},\cdots ,X_{M}^{\left(s\right)}\right\}}_{s=1}^{N_{s}}$ for each participant, where *M* is the number of trials of a SSVEP target, (s) is the index of the SSVEP target, and $N_{s}$ is the number of SSVEP targets (or classes). Each $X_{m}^{\left(s\right)},m=1,\cdots ,M,s=1,\cdots ,N_{s}$ is a matrix of $N_{ch}\times N_{t}$, where $N_{ch}$ is the number of channels and $N_{t}$ the number of samples. Additionally, we assume that the multi-channel EEG signals are centralized since, in practice, the EEG trials are bandpass filtered or detrended.

### 2.2. Canonical Correlation Analysis (CCA)

Spatial filtering attempts to maximize the SNR between the raw EEG data and the their spatially filtered version. In typical cases, such as bipolar combination or Laplacian filtering, the spatial filters are determined manually. However, this approach does not take into account any prior knowledge about SSVEPs or any subject-specific information. One of the first approaches that takes into consideration the structure of SSVEPs was based on Canonical Correlation Analysis (CCA) [20]. CCA is a multivariate statistical method attempting to discover underlying correlations between two sets of data [20,38]. These two sets of data are assumed to be only a different view (or representation) of the same original (hidden) data. More specifically, CCA finds a linear projection for each set such that these two sets are maximally correlated in the hidden (dimensionality-reduced) space.

In the SSVEP problem, these two views are the test EEG trial $X_{m}^{\left(s\right)}$ and the reference templates for *s*-th stimulus $Y_{\left(f_{s}\right)}$, where

$$Y_{\left(f_{s}\right)}={\left[\begin{array}{c} \sin(2\pi \cdot 1\cdot f_{s}t)\\ \cos(2\pi \cdot 1\cdot f_{s}t)\\ \vdots \\ \sin(2\pi \cdot N_{h}\cdot f_{s}t)\\ \cos(2\pi \cdot N_{h}\cdot f_{s}t) \end{array}\right]}^{⊤}$$

$Y_{\left(f_{s}\right)}\in R^{N_{t}\times 2N_{h}}$, $f_{s}$ is the frequency of *s*-th stimulus

Typically, CCA methods maximize the linear correlation between the projections $w_{s}^{T}X_{m}^{\left(s\right)}$ and $v_{s}^{T}Y_{f_{s}}$, where $w_{s}\in R^{N_{ch}}$ and $v_{s}\in R^{N_{t}}$. At the end, we solve the following optimization problem:

(1) $$\begin{array}{c} \max\rho_{s}=\max_{w_{s},v_{s}}\frac{w_{s}^{⊤}X_{m}^{\left(s\right)}Y_{f_{s}}^{⊤}v_{s}}{\sqrt{w_{s}^{⊤}X_{m}^{\left(s\right)}{\left(X_{m}^{\left(s\right)}\right)}^{⊤}w_{s}v_{s}^{⊤}Y_{f_{s}}Y_{f_{s}}^{⊤}v_{s}}} \end{array}$$

Since $\rho_{s}$ is invariant to the scaling of $w_{s}$ and $v_{s}$, the above optimization problem can be also formulated as the following generalized eigenvalue problem:

(2) $$\begin{array}{c} X_{m}^{\left(s\right)}Y_{f_{s}}^{⊤}{\left(Y_{f_{s}}Y_{f_{s}}^{⊤}\right)}^{-1}Y_{f_{s}}{\left(X_{m}^{\left(s\right)}\right)}^{⊤}w_{s}=\lambda_{s}X_{m}^{\left(s\right)}{\left(X_{m}^{\left(s\right)}\right)}^{⊤}w_{s} \end{array}$$

where $\lambda_{s}$ is the eigenvalue corresponding to the eigenvector $w_{s}$. In order to find the stimulus of the test EEG trial, $X_{m}^{\left(s\right)}$, that the subject desires to select, we find features $\rho_{s}$ for all available stimuli, and then, the stimulus-target, *c*, is identified by finding the index of the maximum feature among $N_{s}$ features: $c=\arg\max_{s}\left\{\rho_{s}\right\}$. It must be observed here that there is no need for training (or calibration) since the templates $Y_{f_{s}}$ are artificially generated.

### 2.3. Task-Related Component Analysis (TRCA)

Task-related component analysis (TRCA) enhances the reproducibility of SSVEPs across multiple trials and the intuition of TRCA is to maximize the reproducibility of SSVEP target-related components after spatial filtering. More specifically, the TRCA method finds the spatial filters $w_{s}$ by solving a generalized eigenvalue problem, which is described by the following equation:

(3) $$\begin{array}{c} \max_{w_{s}}\frac{w_{s}^{⊤}AA^{⊤}w_{s}}{w_{s}^{⊤}BB^{⊤}w_{s}} \end{array}$$

where $A=\frac{1}{M}\sum_{m=1}^{M}X_{m}^{\left(s\right)}$, and *B* is a concatenated matrix containing all trials of *s*-th stimulus, $B=[X_{1}^{\left(s\right)},X_{2}^{\left(s\right)},\cdots X_{M}^{\left(s\right)}]$.

Spatial filtering provides us with a more suitable signal for discrimination purposes and not with a classification rule. A widely used rule to discriminate SSVEP responses after spatial filtering is based on correlations of spatial-filtering-derived EEG signals [24]. More specifically, in order to find the target of the test trial, $X_{test}$, we apply the following discriminant function:

(4) $$\begin{array}{c} c=\arg\max_{s}\left\{corr\left(w_{s}^{⊤}X_{test},w_{s}^{⊤}A\right)\right\} \end{array}$$

where corr(·,·) denotes the Pearson’s correlation coefficient.

### 2.4. Adaptive Task-Related Component Analysis (adTRCA)

In our work, we propose a new generalized eigenvalue problem for SSVEP detection, which is described by the following equation:

(5) $$\begin{array}{c} \max_{w_{s}}\frac{w_{s}^{⊤}ACA^{⊤}w_{s}}{w_{s}^{⊤}BDB^{⊤}w_{s}} \end{array}$$

where *C* and *D* are “filtering” matrices that act on the time dimension of the trials. The matrices *C* and *D* can be defined using various approaches, and their goal is to remove noise in time domain.

In our study, we make some critical assumptions about the generation model of SSVEP responses, which affect the data analysis procedure. More specifically, SSVEP responses contain strong sinusoidal components [20]; hence, the SSVEP signal in each channel is modeled as a linear combination of sinusoids described the following matrix:

$$\begin{array}{c} \Phi =\left[Y_{\left(f_{1}\right)}Y_{\left(f_{2}\right)}\cdots Y_{\left(f_{N_{s}}\right)}\right]\in R^{N_{t}\times \left(2N_{s}N_{h}\right)}. \end{array}$$

Additionally, SSVEP responses belonging to the same visual stimulus share common components. From the above, we can observe that the generation of SSVEP responses can be modeled as multiple regression tasks that share common information.

EEG trials from the *s*-th stimulus are collected in matrix $B={\left[{X_{1}^{\left(s\right)}}^{⊤},{X_{2}^{\left(s\right)}}^{⊤},\cdots {X_{M}^{\left(s\right)}}^{⊤}\right]}^{⊤}$, $B\in R^{N_{t}\times \left(N_{ch}N_{s}\right)}$, where each column of B contains the data from one channel or each column of B contains the data from one task. Hence, we have $y_{i}\in R^{N_{t}\times 1},i=1,\cdots ,N_{ch}N_{s}$ tasks (the *i*-th column of B). Each learning task can be described by the following linear regression model:

(6) $$\begin{array}{c} y_{i}=\Phi w_{i}+e_{i} \end{array}$$

where $w_{i}$$2N_{s}N_{h}\times 1$ is a vector of weights (or parameters), and $e_{i}$$N_{t}\times 1$ is a vector of noise coming from a zero mean Gaussian random variable with unknown precision (inverse variance) $a_{0}$. We can observe that each of the mappings yield a corresponding regression task, and performing multiple of such learning tasks has been referred to as multi-task learning [39], which aims at sharing information effectively among multiple related tasks. In a more abstract view of our problem, we can see that each learning task is a linear regression problem, and sinusoidal components from one regression task affect the fitting procedure of another regression task.

The likelihood function for parameters $w_{i}$ and $a_{0}$ is given by:

(7) $$\begin{array}{c} p\left(y_{i}|w_{i},a_{0}\right)={\left(2\pi a_{0}\right)}^{-\frac{N_{t}}{2}}\exp\left\{-\frac{a_{0}}{2}{∥y_{i}-\Phi w_{i}∥}_{2}^{2}\right\} \end{array}$$

The parameters of a regression task, $w_{i}$, are assumed to be drawn from a product of zero-mean Gaussian distributions that are shared by all tasks. Letting $w_{i,j}$ be the *j*-th parameters for *i*-th task, we have:

(8) $$\begin{array}{c} p\left(w_{i}|a\right)=\prod_{j=1}^{2N_{s}N_{h}}N\left(w_{i,j}|0,a_{i}^{-1}\right) \end{array}$$

where the hyperparameters $a={\left\{a_{j}\right\}}_{j=1,2,\cdots ,2N_{s}N_{h}}$ are shared among $N_{ch}N_{s}$ regression tasks; hence, data from all regression tasks contribute to learning these hyperparameters. To promote sparsity over parameters, we place Gamma priors over hyperparameters a [39,40]. In addition, the same type of priority is placed over noise precision $a_{0}$:

(9) $$\begin{array}{cc} p(a_{0}|\alpha ,\beta ) & =Ga(a_{0}|\alpha ,\beta )\\ \; & =\frac{\beta^{\alpha }}{\Gamma (\alpha )}a_{0}^{\alpha -1}\exp\left\{-\beta a_{0}\right\} \end{array}$$

(10) $$\begin{array}{c} p\left(a|c,d\right)=\prod_{j=1}^{2N_{s}N_{h}}Ga\left(a_{j}|c,d\right) \end{array}$$

In addition, we can observed here that noise properties are shared among different tasks (i.e., the noise vectors in Equation (6) are drawn from the same Gaussian distribution). Finally, it must be noted that we have a hierarchical model, and these types of models are natural to be “dealt” with within the Bayesian framework.

Given hyperparameters a and noise precision $a_{0}$, we can apply Bayes’ theorem to find the posterior distribution over $w_{i}$, which is a Gaussian distribution:

(11) $$\begin{array}{cc} p(w_{i}|y_{i},a,a_{0}) & =\frac{p\left(y_{i}|w_{i},a_{0}\right)p\left(w_{i}\right)\left|a\right)}{\int p\left(y_{i}|w_{i},a_{0}\right)p\left(w_{i}\right)\left|a\right)dw_{i}}\\ \; & =N\left(w_{i}|\mu_{i},\Sigma_{i}\right) \end{array}$$

where

(12) $$\begin{array}{cc} \mu_{i} & =a_{0}\Sigma_{i}\Phi^{T}y_{i} \end{array}$$

(13) $$\begin{array}{cc} \Sigma_{i} & ={\left(a_{0}\Phi^{T}\Phi +A\right)}^{-1} \end{array}$$

and $A=diag(a_{1},a_{2},\cdots ,a_{M})$.

In order to find hyperparameters a and promote sparsity in parameters, the type-II Maximum Likelihood procedure is adopted [40,41], where the objective is to maximize the marginal likelihood (or its logarithm). In addition, a similar procedure is followed for noise precision. The marginal likelihood $L(a,a_{0})$ is given by:

(14) $$\begin{array}{cc} \; & L\left(a,a_{0}\right)=\sum_{i=1}^{L}\log\int p\left(y_{i}|w_{i},a_{0}\right)p\left(w_{i}|a\right)dw_{i}\\ \; & =-\frac{1}{2}\sum_{i=1}^{L}\left(N_{i}\log\left(2\pi \right)+\log\left|C_{i}\right|+y_{i}^{T}C_{i}^{-1}y_{i}\right) \end{array}$$

where $C_{i}=a_{0}^{-1}I+\Phi A\Phi^{T}$

Differentiating $L(a,a_{0})$ with respect to a and $a_{0}$ and setting the results into zero [39,40,41] (after some algebraic manipulations) we obtain:

(15) $$\begin{array}{cc} a_{j}^{\left(new\right)} & =\frac{\left(N_{ch}N_{s}\right)-a_{j}\sum_{i=1}^{\left(N_{ch}N_{s}\right)}\Sigma_{i,(j,j)}}{\sum_{i=1}^{\left(N_{ch}N_{s}\right)}\mu_{i,j}},j=1,2,\cdots ,2N_{s}N_{h} \end{array}$$

(16) $$\begin{array}{cc} a_{0}^{\left(new\right)} & =\frac{\sum_{i=1}N_{ch}N_{s}\left(N_{t}-2N_{s}N_{h}+\sum_{j=1}^{2N_{s}N_{h}}a_{j}\Sigma_{i,(j,j)}\right)}{\sum_{i=1}^{N_{ch}N_{s}}{∥y_{i}-\Phi_{i}\mu_{i}∥}_{2}^{2}} \end{array}$$

where $\mu_{i,j}$ is the *j*-th element of $\mu_{i}$ and $\Sigma_{i,(j,j)}$ is the *j*-th diagonal element of the covariance matrix $\Sigma_{i}$. The above analysis suggests an iterative algorithm that iterates between Equations (12), (13), (15), and (16), until a convergence criterion is satisfied. In addition, the same algorithm can be derived by adopting the EM framework and treating parameters $w_{i}$ as hidden variables [40]. Finally, based on the above Bayesian formulation, we can derive a fast version of the above algorithm. The fast version provides an elegant treatment of feature vectors by adaptively constructing the matrix Φ through three basic operators: addition, deletion, and re-estimation. More information on this subject can be found in [39,40].

Now, SSVEP components in each task can be represented as:

$${\hat{y}}_{i}=\Phi \mu_{i},i=1,\cdots ,N_{ch}N_{s}$$

rearranging filtered EEG signals, ${\hat{y}}_{i}$, each filtered EEG trial is represented as: $X_{m}^{f(s)}=X_{m}^{\left(s\right)}a_{0}\Phi \Sigma_{i}\Phi^{⊤}$. Due to filtered trials, we find the spatial filters $w_{s}$ by solving the following generalized eigenvalue problem:

(17) $$\begin{array}{c} \max_{w_{s}}\frac{w_{s}^{⊤}A_{f}A_{f}^{⊤}w_{s}}{w_{s}^{⊤}B_{f}B_{f}^{⊤}w_{s}} \end{array}$$

where $A_{f}=\frac{1}{M}\sum_{m=1}^{M}X_{m}^{f(s)}$, and $B_{f}$ is a concatenated matrix contains all trials of *s*-th stimulus, $B_{f}=\left[X_{1}^{f(s)},X_{2}^{f(s)},\cdots X_{M}^{f(s)}\right]$. The above-generalized eigenvalue problem can be connected by that of Equation (5). After some algebraic manipulations, Equation (17) can be written as:

(18) $$\begin{array}{c} \max_{w_{s}}\frac{w_{s}^{⊤}ACA^{⊤}w_{s}}{w_{s}^{⊤}BDB^{⊤}w_{s}} \end{array}$$

where $C=\left(a_{0}\Phi \Sigma_{i}\Phi^{⊤}\right){\left(a_{0}\Phi \Sigma_{i}\Phi^{⊤}\right)}^{⊤}$ and $D=\left[\begin{array}{ccc} C & \cdots & 0\\ \vdots & \ddots & \vdots \\ 0 & \cdots & C \end{array}\right].$ We can observe an interesting connection between the proposed method and the TRCA. When C=I, where I is the unitary matrix, the proposed approach degrades to the TRCA method. We see that the TRCA method is a limiting case of the proposed method. In addition, we can observe that matrices *C* and *D* act on the time dimension of the EEG trials; hence, the time samples are treated differently according to the time dimension rather than equally weighted. In addition, we can observe that filters, represented by matrix *C*, are adapted to the statistical properties of the EEG trials. A more detailed comparison of adTRCA with TRCA and CORCA is presented in Table 1. Finally, after finding the spatial filters, to find the target of the test trial, $X_{test}^{f}$ we apply the following discriminant function:

(19) $$\begin{array}{c} c=\arg\max_{s}\left\{corr\left(w_{s}^{⊤}X_{test}^{f},w_{s}^{⊤}A_{f}\right)\right\} \end{array}$$

### 2.5. Ensemble Case

According to the previous discriminant rule, described by Equation (19), we can observe that to calculate the similarity of the test trial to the stimulus *s*, we first apply spatial filter $w_{s}$. However, since we have enough calibration trials, we can obtain $N_{s}$ spatial filters for each stimulus [24,29]. Hence, we can extend our method using an ensemble approach, similar to [24,29], where all spatial filters for stimulus *s* are concatenated to create an ensemble spatial filter $W_{s}\in R^{N_{ch}\times N_{s}}$. Now, in order to find the target of the test trial, $X_{test}^{f}$ we apply the following discriminant function:

(20) $$\begin{array}{c} c=\arg\max_{s}\left\{corr\left(W_{s}^{⊤}X_{test}^{f},W_{s}^{⊤}A_{f}\right)\right\}, \end{array}$$

where, now, the function corr(·) depicts the correlation between matrices.

## 3. Results

In order to evaluate our approach, we have analyzed two widely used SSVEP datasets, the *Speller* dataset and the *EPOC dataset*. Furthermore, a short description of each dataset is provided in the next paragraphs.

*Speller* dataset [42]: This dataset was created by acquiring SSVEP responses from thirty-five subjects. The visual stimuli were presented into an 23.6-inch monitor, and the number of different visual was 40. Sixty-four channels were used to acquire EEG signals based on an extended 10–20 system. From these channels in our study, we used the nine channels covering the occipital and parietal–occipital areas *(Pz, PO5, PO3, POz, PO4, PO6, O1, Oz, O2)*. Each subject completed six blocks, where, in each block, the subject was looking at the visual stimuli for 5 s. Furthermore, in each block, the subject was looking at 40 different visual stimuli, one for each target. After the extraction of EEG trials, the signals were band-pass filtered from 7 to 90 Hz with an infinite impulse response (IIR) filter using the *filtfilt* Similar to [42], a delay of 140 ms was considered.

*EPOC* dataset [9]: In this dataset, EEG signals, during an SSVEP-based experimental protocol, were acquired using the Emotiv EPOC device, with 14 wireless channels and a sampling rate of 128 Hz. Visual stimuli were flashing at frequencies of: 6.66 Hz, 7.50 Hz, 8.57 Hz, 10.00 Hz, and 12.00 Hz. Each subject completed 20 trials for each of the five targets. The EEG data have been band-pass filtered from 5 Hz to 45 Hz. More information about this dataset can be found in [9] and https://physionet.org/content/mssvepdb/1.0.0/ (accessed on 3 September 2016).

### 3.1. Performance Metrics

In our study, a leave-one-block-out cross-validation scheme is adopted. More specifically, B−1 blocks are used for training and 1 block for testing, similar to [15,16,23,42]. Furthermore, to evaluate the performance of the methods, we use two widely used measures in the BCI community, the classification accuracy and the Information Transfer Rate (ITR) [16]. Accuracy is the ratio between the correctly classified SSVEP targets to the total number of targets. However, in BCI applications, we are interested, in addition to the accuracy, in the number of classes and the used time of EEG signals. The ITR measure takes into account all the above parameters [16].

We compare the proposed spatial filtering method with two spatial filtering approaches, the CCA [20] and the TRCA [24], and with two ML methods, the MLR approach [15] and the Graph-based Sparse Representations Classification (MLR-SRC) [43]. We calculate the above metrics with respect to the time window of the test EEG trial, the number of EEG channels, and the number of training trials.

### 3.2. Performance Comparison versus the Time Window Length

**Figure 1:**
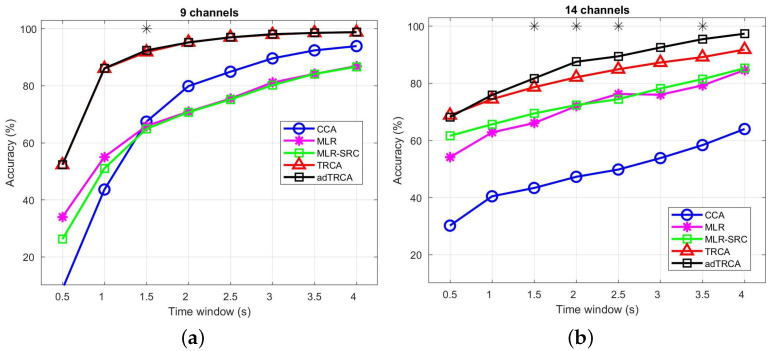
Average Classification over all subjects (**a**) for the Speller dataset and (**b**) the EPOC dataset 14 using the basic configuration with respect to the EEG channels. In both cases, the time window ranges from 0.5 s to 4 s (0.5 s interval). * indicates statistically significant difference between the TRCA and adTRCA methods, using paired sample *t*-test for Speller dataset and Wilcoxon signed rank test for the EPOC dataset (p<0.05).

In the first series of experiments, we follow a typical analysis of SSVEP datasets by examining the performance of methods with respect to the time window. More specifically, the performances of the methods are evaluated for variable times from 0.5 s to 4 s with a step of 0.5 s. In addition, for the Speller dataset, we use the nine channels from the occipital and parietal–occipital areas *(Pz, PO5, PO3, POz, PO4, PO6, O1, Oz, O2)*, while, for the EPOC dataset, we use all 14 available channels, covering the entire brain. In Figure 1, we provide the obtained results for all comparative methods for the two datasets using the basic channel configuration. We can observed the adTRCA and TRCA methods provide better results than all other methods in both datasets. In addition, we can observed that between the adTRCA and TRCA, the adTRCA method has marginally better detection accuracy in the *Speller* dataset and significantly better detection accuracy in the *EPOC* dataset. Similar conclusions can be drawn with respect to the ITR (see Table 2 and Table 3). We can observe that, for most time windows (TWs), the adTRCA methods provide the best ITR among all methods. However, we must point out that for the *Speller* dataset, the best ITR is achieved at TW = 0.5 s from the adTRCA method, while, for the *EPOC* dataset, the best ITR value is achieved at TWs = 0.5 s from the TRCA method.

### 3.3. Performance Comparison Using the Minimal Number of EEG Channels

In the second series of experiments, we have used the minimal number of EEG channels to evaluate the performance of the methods. These EEG channels covering the occipital area (the main brain area in which SSVEPs are present) depend on the EEG device that had been used in each dataset. More specifically, for the *Speller* dataset, *O1, O2*, and *Oz* EEG channels are used, while for the *EPOC* dataset, we use the *O1* and *O2* channels. Note here that the above channel selection procedure is not random; rather, it was performed by taking into account the used EEG devices as well as the International 10–20 system for the placement of EEG electrodes. This scenario corresponds to cases where we are not able to use high-density EEG devices, such as in BCI applications outside a controlled environment.

**Figure 2:**
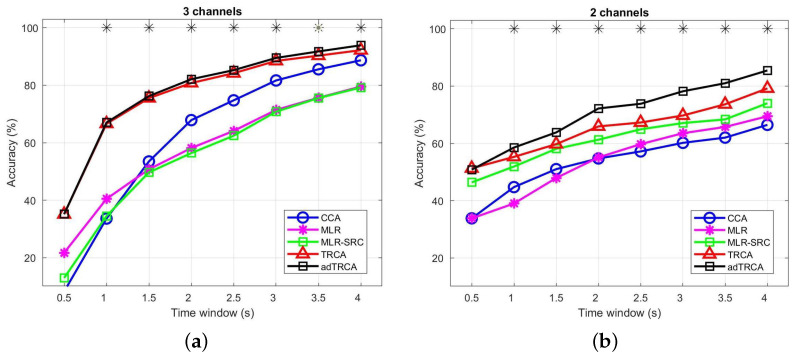
Average Classification over all subjects (**a**) for the Speller dataset and (**b**) for the EPOC dataset using the EEG channels covering the occipital areas. In both cases, the time window ranges from 0.5 s to 4 s (0.5 s interval). * indicates statistically significant difference between the TRCA and adTRCA methods, using paired sample *t*-test for the Speller dataset and Wilcoxon signed rank test for the EPOC dataset (p<0.05).

In Figure 2, we provide the obtained results for all comparative methods for the two datasets using the minimal channels’ configuration. We can observe that the adTRCA and TRCA methods provide better results than all other methods in both datasets. In addition, we can observed that between the adTRCA and TRCA, the adTRCA method has significantly better detection accuracy in both datasets. In Table 4 and Table 5, we provide the results with respect to the ITR measure for all methods. We can observe that for all TWs, except one, the adTRCA methods provides the best ITR among all methods. However, while for the *Speller* dataset the best ITR was achieved at TW = 0.5 s from the adTRCA method, for the *EPOC* dataset, the best ITR value is achieved in the same TW by the TRCA method.

### 3.4. Performance Comparison versus the Number of Training Trials

In the last series of experiments, we investigate how the performance of TRCA and adTRCA are affected by the number of training trials when the time window is 1 s. The obtain results are provided in Table 6 and Table 7. The proposed method clearly has better accuracy from TRCA. Additionally, the proposed scheme achieves the best performance among them, and the margin provided by it is more distinct when a smaller number of training blocks are utilized. Especially, in the case of the Speller dataset, when only three training blocks are utilized, the proposed scheme achieves a classification accuracy of 63%, while the TRCA method achieves an accuracy of 61%. Furthermore, in the case of the EPOC dataset, when three training blocks are used, the proposed scheme achieves a classification accuracy of 33%, while the TRCA method achieves an accuracy of 22% (slightly above the random guess).

### 3.5. Ensemble Case—Experiments

**Figure 3:**
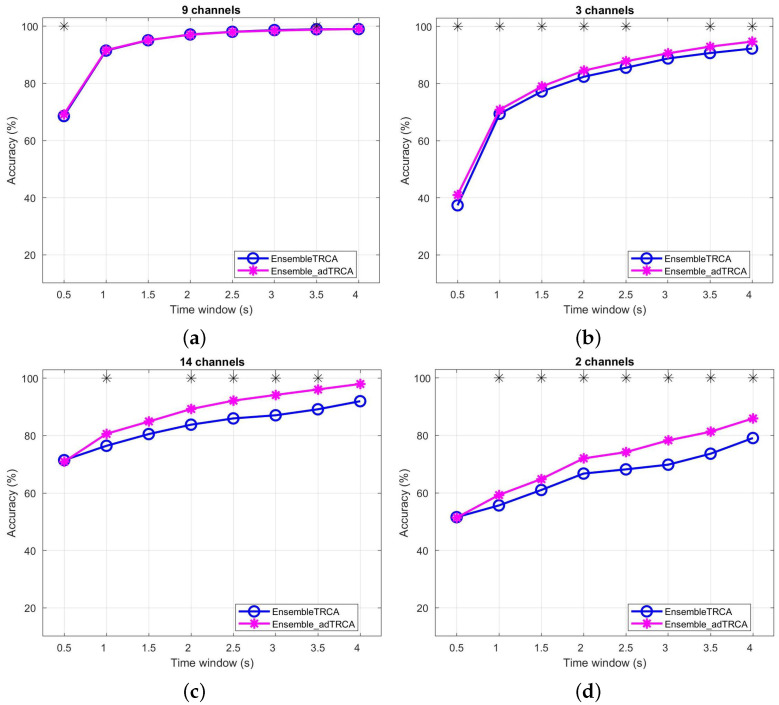
Average Classification over all subjects by using for the Speller dataset with (**a**) 9 channels and (**b**) 3 channels and for the EPOC dataset with (**c**) 14 channels and (**d**) 2 channels, respectively. In both cases, the time window ranges from 0.5 s to 4 s (0.5 s interval). * indicates statistically significant difference between the two methods using paired sample *t*-test for the Speller dataset and Wilcoxon signed rank test for the EPOC dataset (p<0.05).

In the previous subsection, we have presented experiments and we performed comparisons using the basic version of our method. In the current subsection, we provide the obtained results of the ensemble version of our method (see Section 2.5), and we also perform a comparison with ensemble TRCA [24]. More specifically, in Figure 3, we provide the obtained results for the ensemble TRCA (EnsembleTRCA) and ensemble Adaptive TRCA (Ensemble_adTRCA) methods. The comparison between the two methods is performed with respect to the number of channels and the datasets. For the *Speller* dataset, we can observe that in the case of nine channels the two methods present similar performance. However, in the case of three channels, the Ensemble_adTRCA method provides significantly better performance than the EnsembleTRCA method. Additionally, when we use the *EPOC* dataset, the Ensemble_adTRCA method provides significantly better performance, either using all 14 channels of the EPOC device or the 2 channels covering the occipital lobe.

**Figure 4:**
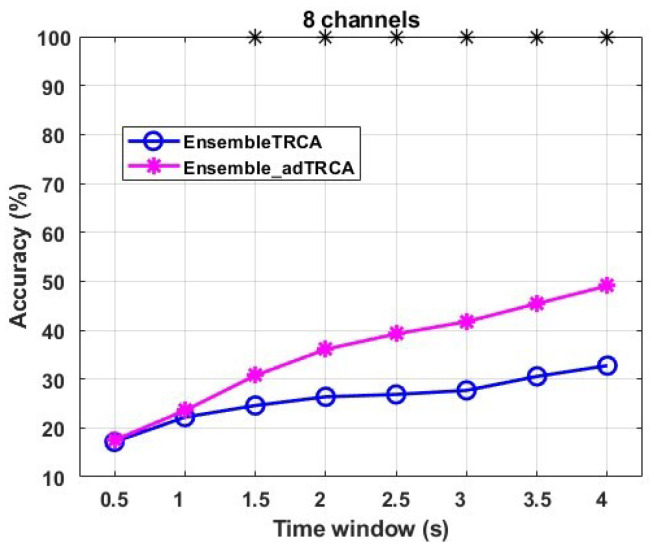
Average Classification in Hairless case for the Speller dataset. The time window ranges from 0.5 s to 4 s (0.5 s interval). * indicates statistically significant difference between the two methods using paired sample *t*-test (p<0.05).

We have performed another experiment using ensemble methods (EnsembleTRCA and Ensemble_adTRCA). This experiment corresponds to the case of using hairless EEG channels (*FP1, FPz, FP2, TP7,* and *TP8*) of the *Speller* dataset. Prefrontal and near-the-ear EEG channels have several attractive properties for real-world applications: discreet (not clearly visible), unobtrusive, comfortable to wear, impeding the user as little as possible, and they are user-friendly since they can be operated and attached by the user [32]. However, there is a compromise in recording quality, resulting in noisy, low SNR SSVEP signals. This experiment has been executed using the SSVEP data from the *Speller* dataset. Additionally, we choose a priori the following stimulation frequencies: 8 Hz, 9 Hz, 10 Hz, 11 Hz, 12 Hz, 13 Hz, 14 Hz, and 15 Hz (an eight-class classification problem). The obtained results are provided in Figure 4. We can observe that the Ensemble_adTRCA method provides better results than the EnsembleTRCA, justifying our assumption that the performance of the typical TRCA approach is deteriorated in noisy environments. More specifically, we can see that the Ensemble_adTRCA provides better classification accuracy from the the EnsembleTRCA, in some cases more than 15%. Furthermore, a comparison of Figure 3 and Figure 4 clearly shows that the performance of both methods is deteriorated comparing to the case of using channels from the occipital lobe.

## 4. Discussion and Conclusions

Enhancing the performance of SSVEP recognition is a significant issue for BCI applications. In this study, we develop a multi-task learning scheme to strengthen the TRCA method. The idea behind the proposed learning scheme is to develop an adaptive time-domain filter which can be used in a more general eigenvalue problem than the corresponding problem of the TRCA method. The proposed method is able to deal with more general noises and with a reduced number of trials, as the experiments have shown. However, this increase in performance from our method has an increase also in the computation time of the overall procedure, since an iterative method is used to find the adaptive time-domain filters.

A significant part of our study is the use of two SSVEP datasets to evaluate our method. Most SSVEP studies use the Speller dataset to evaluate the proposed methods. However, this dataset was created into a controlled environment with high-cost EEG equipment, which makes it difficult to replicate the study for real BCI applications with low-cost equipment and in very noisy environments. Hence, the aforementioned methods that are evaluated on the Speller dataset tend to underestimate the noise part of SSVEP EEG trials, an effect which can be observed by comparing the performance of adTRCA and TRCA on both datasets. We can see that the adTRCA method provides much better performance than the TRCA in the case of the EPOC dataset. In the Speller dataset, the adTRCA method has around 1% better accuracy than the TRCA, while, in the EPOC dataset, this difference is increased to 5%. Additionally, the ensemble version of our method presents better performance than the ensemble version of TRCA in both datasets, especially when we have a limited number of channels. Finally, in the case of using only channels placed in hairless brain regions, the proposed approach provides us with a significantly better performance (more than 15%) than the classical TRCA method.

The spatial filters and the SSVEP templates play important roles in the target recognition methods. When the spatial filters and the SSVEP templates cannot be accurately computed, e.g., in the case of small calibration data or noisy EEG recordings, the resulting recognition performance will be dramatically decreased. Hence, to this challenge, the key is how to estimate reliable spatial filters. In this study, we present a novel spatial filtering approach to recognize SSVEP signals. Our method uses the multi-task idea to construct adaptive time-domain filters resulting into a generalized eigenvalue problem from where the final spatial filters are obtained. Extensive experiments, using two SSVEP datasets, have shown the usefulness of our method. The proposed method significantly outperformed the TRCA, the CCA, the MLR, and the SRC methods in terms of classification accuracy and ITR.

In the future, we intend to examine SSVEP scenarios that will result in BCI systems that are more comfortable and have a better user experience. Prefrontal EEG channels, which have been used successfully in driver drowsiness detection [44], plays significant role in such systems since they are placed in hairless brain regions, and they provides us with signals less subjected to noise [45]. Furthermore, there are indications that occipital and frontal areas play important roles in the generation of SSVEP signals [46]. Hence, adopting these channels for the design of SSVEP BCI systems is an appealing idea. However, care must be taken since the primary activation area of SSVEP responses is the occipital lobe. It is our intention to investigate in the future recognition algorithms by using only prefrontal EEG channels. This requires new pre-processing EEG algorithms to remove eye-blinks and new recognition algorithms since it is expected that the acquired SSVEP response in the prefrontal brain area will have different properties from those in occipital areas. Furthermore, the information flow between occipital and frontal areas during the generation of SSVEP signals is an important factor that must be examined in these kinds of experiments. In addition, future extensions of our approach could include transfer learning approaches utilizing the data from all subjects to construct the recognition model. Recently, SSVEP BCI systems using ear-EEG recordings have been proposed; however, the accuracy of such systems is low compared to classical approaches because the EEG sensors are located far from the occipital cortex, resulting in low SNR in the SSVEPs. Hence, there is a need for new recognition algorithms working in noisy environments.

## Figures and Tables

**Table 1:** Differences in the objective function of the proposed method (adTRCA) with respect to TRCA [24] and CORCA [29].

TRCA	CORCA	Adaptive TRCA (adTRCA)
$S=AA^{⊤}$	$S=R_{11}$	$S=ACA^{⊤}$
$Q=BB^{⊤}$	$Q=R_{12}$	$Q=BDB^{⊤}$

*Note*: *R*_11_ is the intrasubject covariance matrix, *R*_12_ is the intersubject cross-covariance [29]. Objective Function: max_**w**_*s*__ $\frac{w_{s}^{⊤}Sw_{s}}{w_{s}^{⊤}Qw_{s}}$.

**Table 2:** ITR on Speller dataset—9 channels.

TW	CCA	MLR	MLR-SRC	TRCA	adTRCA
0.5	27.3581	141.5439	98.0103	270.9573	**271.3097**
1	101.8150	137.3785	123.1899	260.0963	**260.3216**
1.5	122.8637	118.8840	116.4590	189.4191	**190.8013**
2	116.7986	98.5886	98.1738	**149.0613**	148.8653
2.5	101.4344	85.9023	85.3361	**122.2336**	122.1442
3	90.6986	79.0954	77.8773	103.1242	**103.3462**
3.5	81.1131	71.3472	71.3823	**89.2204**	89.0756
4	72.5963	65.1814	65.0779	78.2199	**78.3041**

**Table 3:** ITR on EPOC dataset—14 channels.

TW	CCA	MLR	MLR-SRC	TRCA	adTRCA
0.5	13.7550	61.4288	81.6831	**113.1879**	109.7278
1	16.4944	45.1581	49.0298	67.8476	**70.7059**
1.5	13.3129	35.7231	37.9279	52.7534	**55.7158**
2	13.6286	31.7665	31.8755	44.0878	**48.9809**
2.5	12.4521	29.7089	28.1211	38.7167	**42.2460**
3	12.0830	25.1981	26.2000	35.0314	**38.0493**
3.5	12.1995	23.8428	24.4427	31.7552	**34.8263**
4	12.6687	23.6196	23.6422	29.8733	**32.1765**

**Table 4:** ITR on Speller dataset—3 channels.

TW	CCA	MLR	MLR-SRC	TRCA	adTRCA
0.5	26.6513	82.8903	39.9318	162.8981	**163.3228**
1	71.6644	93.4390	74.2354	189.5521	**190.6936**
1.5	89.8807	85.1997	82.2758	148.7973	**150.0271**
2	93.5801	76.2840	73.1798	121.8152	**123.6819**
2.5	85.4184	69.7438	67.2738	102.4240	**103.7595**
3	80.0041	67.1743	66.4061	90.6681	**91.6374**
3.5	73.1382	62.4800	62.2848	79.6201	**81.0671**
4	67.1804	58.2207	57.9068	71.3779	**72.9298**

**Table 5:** ITR on EPOC dataset—2 channels.

TW	CCA	MLR	MLR-SRC	TRCA	adTRCA
0.5	20.4758	17.7383	41.2960	**55.2841**	54.7309
1	20.8587	13.2992	29.1648	34.8970	**38.5391**
1.5	20.2541	16.2247	26.1393	29.4451	**31.4028**
2	18.8570	16.9955	21.9714	27.6870	**31.5392**
2.5	16.7189	17.8696	20.5254	23.4503	**27.1407**
3	15.7776	16.6921	18.1873	20.4231	**25.5093**
3.5	14.5984	15.0986	16.8087	19.7346	**23.7536**
4	14.6751	15.3353	17.0919	19.9582	**23.4404**

**Table 6:** Classification accuracy (%) on Speller dataset with respect to the number of training trials—9 channels.

Num	TRCA	adTRCA
3	61.5714	**63.0238**
4	82.9429	**83.3000**
5	82.9429	**83.3000**
6	86.0476	**86.1429**

**Table 7:** Classification accuracy (%) on EPOC dataset with respect to the number of training trials—2 channels.

Num	TRCA	adTRCA
3	22.4242	**33.3333**
7	36.8831	**39.4805**
10	37.6364	**39.8182**
15	42.7879	**42.7879**
20	54.5455	**55.2727**

## Data Availability

The datasets that have been used in this study are available on the Internet. The *Speller* dataset can be found at http://bci.med.tsinghua.edu.cn/download.html (accessed on 9 June 2017). The *EPOC* dataset can be found at https://physionet.org/content/mssvepdb/1.0.0/ (accessed on 3 September 2016).
